# Results of a pilot randomised controlled trial to measure the clinical and cost effectiveness of peer support in increasing hope and quality of life in mental health patients discharged from hospital in the UK

**DOI:** 10.1186/1471-244X-14-30

**Published:** 2014-02-05

**Authors:** Alan Simpson, Chris Flood, Julie Rowe, Jody Quigley, Susan Henry, Cerdic Hall, Richard Evans, Paul Sherman, Len Bowers

**Affiliations:** 1 School of Health Sciences, City University London, Northampton Square, London EC1V 0HB, UK; 2 KentHealth, Centre for Health Services Studies, University of Kent, Canterbury, Kent CT2 7NF, UK; 3 School of Psychological Sciences and Health, University of Strathclyde, Graham Hills Building, 40 George Street, Glasgow G1 1QE, UK; 4 East London NHS Foundation Trust, 22 Commercial Street, London E1 6LPUK, UK; 5 Institute of Psychiatry, King’s College, London SE5 8AF, UK

**Keywords:** Peer support, Mental health, Discharge, Hope, Loneliness, Quality of life, Economic evaluation, Suicide

## Abstract

**Background:**

Mental health patients can feel anxious about losing the support of staff and patients when discharged from hospital and often discontinue treatment, experience relapse and readmission to hospital, and sometimes attempt suicide. The benefits of peer support in mental health services have been identified in a number of studies with some suggesting clinical and economic gains in patients being discharged.

**Methods:**

This pilot randomised controlled trial with economic evaluation aimed to explore whether peer support in addition to usual aftercare for patients during the transition from hospital to home would increase hope, reduce loneliness, improve quality of life and show cost effectiveness compared with patients receiving usual aftercare only, with follow-up at one and three-months post-discharge.

**Results:**

A total of 46 service users were recruited to the study; 23 receiving peer support and 23 in the care-as-usual arm. While this pilot trial found no statistically significant benefits for peer support on the primary or secondary outcome measures, there is an indication that hope may be further increased in those in receipt of peer support. The total cost per case for the peer support arm of the study was £2154 compared to £1922 for the control arm. The mean difference between costs was minimal and not statistically significant. However, further analyses demonstrated that peer support has a reasonably high probability of being more cost effective for a modest positive change in the measure of hopelessness. Challenges faced in recruitment and follow-up are explored alongside limitations in the delivery of peer support.

**Conclusions:**

The findings suggest there is merit in conducting further research on peer support in the transition from hospital to home consideration should be applied to the nature of the patient population to whom support is offered; the length and frequency of support provided; and the contact between peer supporters and mental health staff. There is no conclusive evidence to support the cost effectiveness of providing peer support, but neither was it proven a costly intervention to deliver. The findings support an argument for a larger scale trial of peer support as an adjunct to existing services.

**Trial registration:**

Current Controlled Trials ISRCTN74852771

## Introduction

Mental health patients recently discharged from hospital often fail to continue with treatment [1], relapse and are readmitted [2]. In the UK, between 20% and 40% of psychiatric patients were reported to be re-admitted within six months of discharge with the peak period occurring within the first month [3]. Suicide is also a risk with around 8% of all community suicides occurring in the two weeks following discharge [4]. Research interviews with service users on mental health units found that over half said they felt anxious about being discharged [5]. In hospital, they felt safe and supported by staff and appreciated the company of other patients and were concerned about coping outside. One suggestion to improve post-discharge outcomes was the addition of peer support by fellow service users alongside existing aftercare services [5].

## Background

Formal peer support (PS) consists of social and emotional support provided by people with experience of illness to others sharing a similar condition to bring about a desired social or personal change [6]. It has been suggested that peer support workers (PSWs), who have achieved significant recovery of their own, offer acceptance, respect, empathy, support, companionship, hope and share experiences and ideas about how to cope with mental illness [7].

Peer support programmes have been developed and incorporated into state run services in the USA [8] and fledging services are operating in Scotland and England [9,10]. A broad review of the international literature on PS found evidence supporting its effectiveness in empowering service users, aiding recovery and in a small number of studies, reducing re-admissions [11]. Most studies reported potential benefits both for service users and PSWs, and may increase hope, improve social networks and aid recovery [12-15].

A recent Cochrane Review assessed the effects of employing consumers of mental health services as providers of services in roles that included peer support, coaching, advocacy, case management and outreach or crisis worker [16]. Five trials involving 581 people compared consumer-providers to professionals in similar roles and found no significant differences across a wide range of measures; consumer providers were as effective as professionals. There was a small reduction in crisis and emergency service use for clients receiving care involving consumer-providers. Consumers who provided mental health services did so differently than professionals; they spent more time face-to-face with clients, and less time in the office, on the telephone, with clients’ friends and family, or at provider agencies. Six trials involving 2215 people compared mental health services with or without the addition of consumer-providers. Again, there were no significant differences across various measures between groups with consumer-providers as an adjunct to professional-led care and those receiving usual care from health professionals alone. The quality of studies was moderate, most undertaken in the USA and cost effectiveness was not considered.

In Canada and Scotland, trials of a transitional discharge model in which support was provided at discharge jointly by ward staff and PSWs, reported reductions in re-admissions and use of emergency services, lower costs and increased satisfaction [17-19]. In Australia, a three-month pilot study of PS aimed at reducing hospital admissions reported significant reductions in admissions and re-admissions, less use of emergency services and associated cost savings [20]. No trials have been conducted in the UK into the effectiveness of peer support as an intervention for patients at the transition point of being discharged from hospital.

PS is considered to be potentially cost effective because of the possible reduction in hospital admissions and other resource use. The clinical effectiveness and cost effectiveness of peer support is an important research question. There are potential benefits for the wellbeing of patients at a difficult transitional stage and potential costs savings for the health care system that can then allow resources to be used elsewhere in the care system.

### Aims and objectives

In this pilot study, we aimed to:

1. Investigate the effect of peer support on feelings of hope and loneliness, quality of life and service use in mental health patients following discharge from hospital.

2. Evaluate the economic consequences of the intervention.

3. Inform the design of a definitive RCT by:

a. providing realistic estimates of recruitment and retention;

a. assessing the feasibility of the methods of randomisation including acceptability to participants;

a. providing information on the limitations of implementation;

a. providing data to inform future sample size calculations, intra-class correlations and descriptive statistics on the baseline performance of the final outcome measures.

It was hypothesised that peer support would have a positive effect on the patients’ feelings of hope, reduce their loneliness and improve quality of life with an effect on service use, including reduced re-hospitalisation, and decrease the risk of suicide. The economic evaluation also sought to explore the relative cost-effectiveness of peer support services compared with care as usual, comparing service use data and associated costs collected at three month follow-up. Such information is important as it enables policy and decision makers to compare the costs incurred and services used by recipients of the peer support intervention as compared to those receiving standard care. The study also aimed to conduct a cost utility analysis.

## Methods

### Design

Pilot randomised controlled trial (RCT) with economic evaluation comparing peer support in addition to care as usual with usual care alone following discharge, with follow-up at one month and three months post-discharge.

### Intervention condition

PSWs to provide peer support for four weeks to patients discharged from four mental health wards. Initial contact was made while the patient was an inpatient with discharge expected within the next two to three weeks (total contact time six weeks). Peer support would be in addition to care as usual (CAU).

### Control condition

Patients in the control condition received CAU arrangements from community mental health services.

### Inclusion criteria

Diagnosed mental illness; approaching discharge/extended leave; age 18–65.

### Exclusion criteria

Considered a serious risk to others; alcohol or drug dependent or primary diagnosis of substance use; serious personality disorder; pregnant or caring for children.

### Sample size and power calculation

The Beck Hopelessness Scale (BHS) is the main outcome measure [21]. A power calculation was undertaken, based on the results of a study of 400 randomly selected adults from the general population that reported a mean of 4.45 with a standard deviation of 3.09 [22]. Using these norms, we calculated that a sample size of 39 in each group (total sample 78) would have 80% power to detect a difference in means of −2.000 (the difference between a Group 1 mean of 4.450 and a Group 2 mean of 6.450) assuming that the common standard deviation is 3.090 using a two group *t*-test with a 0.050 two-tailed significance level [23,24].

### Sampling rationale

Previous research reported an average 60 patients per month discharged from the four wards in inner London, England due to take part in this study [25]. Allowing for patients swiftly discharged from the ward, exclusion of those not meeting inclusion criteria, and others that declined to take part, we aimed to recruit 110 service users into the trial over seven months at a rate of approximately 16 per month (26% of the predicted discharges). With 55 patients in each arm of the trial, we allowed for a 10 per cent drop-out rate before the end of the intervention and again before the three-month follow-up, providing 45 users in each arm, a total of 90 participants.

**Figure 1:**
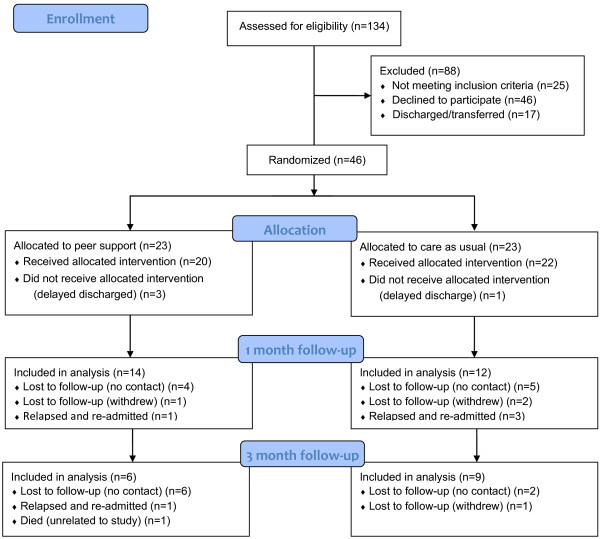
CONSORT flow diagram.

Recruitment and follow-up: A total of 46 patients were recruited to the study; 23 receiving peer support and 23 in the care-as-usual arm (see Figure 1). At one-month (T1) follow-up, 26 participants (56.5%) completed data collection (14 PS; 12 CAU); and at three-month (T2) follow-up, 15 (36.2% of the total sample; 57.7% T1 completers) completed data collection (6 PS; 9 CAU).

### Randomisation

Randomisation was by ‘distance’ randomization using a computer programme with no information about the recruited individual [26]. ‘Block’ rather than simple randomization was employed to ensure equal-sized treatment groups [27].

### Data collection and instrumentation

Data was collected at baseline (T0), one month following discharge (T1) and three months following discharge (T2). The following instruments were employed:

a) Patient Data Pro-forma: Demographics, diagnosis and history via case note audit.

b) Beck Hopelessness Scale (BHS) [21] is a 20-item scale for measuring negative attitudes about the future based on three dimensions of hopelessness. It is the main outcome measure as it was hypothesised that PS would increase hope and the scale is established as a reliable measure of suicide risk in psychiatric patients in hospital and outpatient settings [28,29] and with high internal and test-retest reliability [21,30].

c) UCLA Loneliness Scale (V3) [31]. Developed to assess subjective feelings of loneliness or social isolation and modified to increase reliability and validity, the scale is the most widely used measure in studies of loneliness and social isolation with scores predicting a variety of mental and physical health outcomes [31]. Addition of PS is hypothesised to lead to an increase in social networks and reduce loneliness in participants.

d) EuroQol (EQ-5D) Quality of Life Questionnaire is a non-disease-specific instrument for describing and valuing health-related quality of life, used in economic evaluation [32], including alongside RCTs [33]. The EQ-5D descriptive system comprises five dimensions: mobility, self-care, usual activities, pain/discomfort and anxiety/depression. Each dimension is scored: no problems (1); some problems (2); severe problems (3). The EQ VAS records the respondent’s self-rated health on a vertical, visual analogue scale where the endpoints are labelled ‘Best’ and ‘Worst’ ‘imaginable health state’. This information can be used as a quantitative measure of health outcome as judged by the individual respondents. A higher score represents a better perceived health state.

e) Client Service Receipt Inventory (CSRI) was used to collect service use for all service users and in turn calculate costs [34]. Data about health and social services resource use, including hospital admission, was collected from patients, care co-ordinators and clinical records. A data collection pro forma was used to record services provided and number of contacts with each, for all services including peer support.

f) Peer Support Activity Diaries, maintained by PSWs to record the time spent and activities undertaken in support of each user, as an indicator of the type and level of support provided.

### Public participation

The idea for a ‘buddy system’ or peer support on discharge was suggested by the service user researcher on a previous study [35]. Design of the study, ethics application, recruitment and selection of PSWs, progress updates and results were all discussed with members of a service user and carer research advisory group (SUGAR). Three service users and a carer were also members of the Project Advisory Group.

### Procedure

PSWs were recruited (n = 16), with eight successfully completing training and working as PSWs. They were provided with supervision and support for the duration of the study. Potential study participants expected to be discharged within two-to-three weeks were identified by the Peer Support Coordinator (PSC) in discussion with ward staff and invited to take part in the study. Participants completed a consent form and baseline measures administered by the research assistant. Each participant was then randomly assigned to either the experimental (peer support and usual aftercare) or care as usual (CAU) condition and informed. Patients allocated to peer support were then introduced to a PSW by the PSC prior to discharge in order to negotiate peer support lasting a maximum of four weeks following discharge.

### Data collection and analysis

Coded, anonymous data on patient demographics, diagnosis and history were recorded in SPSSv17 and checked for errors and omissions. In line with recommendations for pilot trials [36] the analysis provides means, medians and confidence interval estimations to inform further studies. Relatively small datasets due to lower than expected recruitment and follow-up combined with skewed data determined the use of non-parametric tests to explore scores on the outcome measures.

Costs of services used by each participant were estimated from the quantities of each type of resource used multiplied by the unit cost. Unit costs of resources was derived from routine sources locally where possible, and from national sources judged representative of local costs. Costs associated with the different resources and services used are reported as part of this economic evaluation. To calculate more specifically the costs, we looked at resource use for primary care, which included an examination of total drugs used, visits to general practitioners, dentists and physiotherapy. Secondary care resource use included collecting data for attendance at emergency departments for non-psychiatric care. Mental health service resource use included multidisciplinary staff from community, home treatment crisis resolution and assertive outreach teams. Costs for the year 2010 were used [37].

Included as part of the resource use measurements were the peer support worker visits and contacts based on PSW records kept during each peer support period. Participants’ use of counsellors, therapist visits, housing support worker visits, visits to community mental health centres, day hospital attendances and use of telephone crisis lines were also recorded. Resources associated with psychiatric admissions were recorded and totalled with all the previously identified resources, to calculate a total cost to the health service overall. Resources used and costs to the criminal justice system, with the latter being based on police contact and police doctor contact, were also sought.

A total cost per case per patient was calculated based on all of the above which in turn allowed for an average cost per case per trial arm for treatment and the control group, presented with rates of significance of difference between arms.

A secondary cost utility analysis was performed combining the cost data with the Beck Hopelessness Scale (BHS) data and EuroQol (EQ5D) data. As part of this analysis, non-parametric bootstrapping [38] was used to develop confidence intervals around the incremental cost effectiveness ratio based on costs and effects. This process also generates acceptability curves to illustrate the uncertainty associated with the estimate of costs and effects combined and estimates of affordability given potentially different decision maker cost thresholds.

## Results

A comparison of patient-related factors was undertaken between intervention and control groups to identify any baseline differences between groups (see Table 1). There were no statistically significant differences between age in the PS condition (M = 34.13, SD = 10.27) and the care as usual (CAU) condition (M = 36.36, SD = 10.15), t(44) = −.0742, P = 0.462. The distribution of gender was equal in each group (*χ*2(1) = 1.627, P = 0.202) and both groups contained participants from a wide range of ethnic backgrounds with a broadly similar spread across groups. The two groups were also similar regarding housing status (predominantly council/housing association/supported accommodation or no fixed abode). Those in the peer support group appeared slightly more likely to live alone but this was uncertain given the high level of missing data for this category.

**Table 1:** Participant demographic and baseline characteristics by group

**Characteristics**		**Peer support (n-23)**	**Care-as-usual (n = 23)**
Female		7 (30.4 %)	3 (13.0 %)
Male		16 (69.6 %)	20 (87.0 %)
Age - mean years (sd)		34.13 (10.27)	23.36 (10.15)
Age - range		20-55	22-57
Ethnicity	Black African	7	2
	Black Caribbean	1	7
	Black other	5	2
	Mixed race	4	1
	White UK/Irish	5	7
	White other	1	1
	Chinese	0	1
	Bangladeshi	0	1
Living status	Alone	12 (52.1 %)	14 (60.9 %)
	With others	6	6
	Missing	5	3
Accommodation	Local authority/HA	8 (34.8 %)	8 (34.8 %)
	Supported	5	2
	No fixed address	6	4
	Hostel	0	2
	Own home	1	3
	Rented home	1	3
	Family home	2	0
	Missing	0	1
MHA status	Informal	15 (65.2 %)	14 (60.9 %)
	Formal	7	9
	Missing	1	0
Diagnosis (primary)	Paranoid schizophrenia	8 (34.8 %)	7 (30.4 %)
	Depression	6	2
	Bipolar disorder	3	7
	Psychosis	2	1
	Schizophrenia	1	3
	Schizo-affective disorder	1	1
	Personality disorder	1	1
	Unconfirmed	1	1
Admissions last year	None	11	9
	1 to 3	10	11
	Missing	2	3
Total admissions	None	4	6
	1 to 3	11	10
	4 +	7	7
	Missing	1	0

Around two-thirds of the patients in both groups were ‘informal’ patients, rather than detained under the Mental Health Act. The primary diagnoses in both groups were largely spread around a range of psychotic disorders, although there were a higher proportion of people with depression in the peer support cohort (not significant). Around half of the patients in both arms reported several admissions in the previous three years.

PSW activities including face-to-face contact, phone contact, attempted contacts and liaison with mental health and other staff are summarised in Table 2.

**Table 2:** PSW activity

		**No. contacts per peer**	**Total timeper peer (minutes)**	**No. phone contacts per peer**	**Total time per peer (minutes)**	**No. attempted contacts**	**Total time per attempted contact (minutes)**	**No. contacts with staff**	**Total time per staff contact (minutes)**
N	Valid	21	21	21	21	21	21	21	21
	Missing	2	2	2	2	2	2	2	2
Mean		5.6190	50.9005	8.7143	6.4710	2.9048	9.2729	.8571	11.2290
Median		5.0000	50.0000	7.0000	6.0000	1.0000	1.0000	.0000	.0000
Minimum		1.00	15.00	.00	.00	.00	.00	.00	.00
Maximum		15.00	112.50	30.00	13.88	13.00	100.00	7.00	180.00
Percentiles	25	2.5000	28.9300	3.0000	3.5650	.0000	.0000	.0000	.0000
	50	5.0000	50.0000	7.0000	6.0000	1.0000	1.0000	.0000	.0000
	75	8.0000	63.2350	11.5000	10.0000	6.0000	4.1250	1.5000	5.3350

### Results on key outcome measures

#### Beck’s Hopelessness Scale (BHS)

Higher scores on the BHS denote higher levels of hopelessness. There was no significant difference on BHS scores at baseline between the Peer Support (PS) and care-as-usual (CAU) arms (U = 177, P = 0.083). We compared the baseline (T0) scores on the BHS between those who subsequently completed the BHS at one-month follow-up (T1) and those who did not, using the Mann Whitney *U* Test. The mean rank BHS T0 score was higher for the T1 completers than in the T1 non completers, but was not statistically significant (U = 204.5, P = 0.277).

Differences in the BHS scores between three data points within each condition were compared using the Friedman Test. For both PS and CAU the mean rank scores reduce between T0 and T2, as participants become more hopeful, with a larger drop for those in the PS condition, although this fell short of significance *χ*2(2) = 5.810, P = 0.055. For those in the CAU condition there was no statistical difference in hopelessness between baseline and follow up at one month nor at 3 months *χ*2(2) = 0.276, P = 0.871. Finally, there was no statistical difference in hopelessness at T1 between the PS condition and CAU using the Mann Whitney *U* test (U = 70.5, P = 0.494).

#### UCLA Loneliness Scale V3 (UCLA)

Higher scores on the UCLA denote higher levels of loneliness. We compared the baseline (T0) scores on the UCLA between those who subsequently completed the UCLA at one-month follow-up (T1) and those who did not, using the Mann Whitney *U* Test. Whilst the mean rank UCLA T0 score was higher for the T1 completers than in the T1 non completers, this was not statistically significant (U = 236.5, P = 0.724). We then compared the differences in the UCLA scores between three data points within each condition using the Friedman Test. Loneliness scores increased in the PS arm at T1, then decreased overall at T2, whereas for CAU the mean rank loneliness scores increased at both T1 and T2. However, there was no statistical difference in loneliness between baseline and follow up at one month nor at three months for those in the PS condition *χ*2(2) = 0.609, P = 0.738 or for those in the CAU arm *χ*2(2) = 2.250, P = 0.325. Finally, there was no statistical difference in UCLA scores at T1 between the PS condition and CAU using the Mann Whitney *U* test (U = 68, P = 0.432).

### EQ5D EuroQoL Quality of Life Scale (EQ5D)

There was no significant difference between T1completers and T1non completers on the baseline EQ-5D quality of life total dimensions score (U =238.00, P = 0.732), or the baseline health status VAS scores (U =207.00, P = 0.305). Neither was there any statistical difference in quality of life between baseline and follow up at one month nor at three months for those in the PS condition *χ*^2^(2) = 0.667, *P* = 0.717 or for those receiving CAU *χ*^2^(2) = 2.800, *P* = 0.247.

A similar test comparing the differences in the EQ VAS scores between three data points within each condition revealed a statistically significant difference towards improvement in self-reported general health, depending on time period, for the CAU condition *χ*^2^(2) = 10.640, P = 0.005. No statistical differences in self-reported general health was evident for the PS condition *χ*^2^(2) = 0.667, P = 0.717.

To compare differences in the EQ VAS between the three time points within the CAU cohort, *post-hoc* analysis with Wilcoxon Signed-Rank Tests was conducted with a Bonferroni correction applied, resulting in a significance level set at P < 0.017. Median (IQR) perceived effort levels for care-as-usual for the T0, T1 and T2 were 0.66 (0.23 to 0.86), 0.72 (0.57 to 0.95) and 0.81 (0.62 to 1.00), respectively. There were no significant differences between T0 and T1 scores (Z = −1.362, P = 0.173) or between T1 and T2 general health scores (Z = −1.826, P = 0.068) although the latter was approaching significance. However, there was a statistically significant increase in self-reported general health status from baseline to T2 (Z = −2.366, P = 0.018).

There was no statistical difference at T1 for EQ-5D quality of life between the PS condition and CAU using the Mann Whitney *U* test (U = 71, P = 0.977) or for the EQ-5D health status VAS scores (U = 71, p = 0.977).

### Readmissions

Between discharge and one-month follow-up, one patient receiving PS was readmitted to hospital, compared with three in the CAU arm. Another patient in the PS arm was readmitted before three-month follow-up and one person in this arm died from an unrelated medical condition.

### Resource use and cost outcomes

The total cost per case for the PS arm of the study was £2154 compared to £1922 for the control arm as can be seen in Table 3. The mean difference between costs in the trial arms was minimal and not statistically significant. There were no significant differences in costs for primary care, secondary care, mental health services, and criminal justice or for total costs to the health service system when costed overall. Total drugs resource use and costs, part of the primary care overall costs, initially showed a borderline level of significance. However, having established a borderline leveI of significance for drug costs, the baseline data was checked to see if there were any differences in resource use between the groups after randomisation. This was found to be the case with a significant difference in costs for drug use noted already at baseline between trial arms. These analyses was based on the assumption that missing cost data for participants truly was a zero cost. As there was potentially great uncertainty around this assumption, another analysis was conducted where missing cost data for participants was handled using mean imputation methods for missing data. As a result of conducting this analysis the average cost per case of the Peer Support Work arm was £5103 compared to £3321 for the control arm. The mean difference between costs in the trial arms was again not statistically significant.

**Table 3:** Cost per patient by treatment arm (£ Sterling 2010 prices)

		**PSW arm**			**Control arm**						
		**(N = 6)**			**(N = 9)**						
		**Total cost**						**Mean difference**			
**Type of cost**	**Detail of costs**	**Mean**	**(SD)**	**%**	**Mean**	**(SD)**	**%**	**(PSW - control)**	**(95% CI)**		**P**
Primary care		**126**	**703**	**6**	**317**	**423**	**16**	**191**	**418**	**−37**	**0.11**
	Total drugs	38	117	2	160	257	8	122	252	−7	0.07
	General practitioner	88	211	4	147	187	8	58	186	−70	0.38
	Dentist	0	0	0	4	15	0	4	11	−3	0.31
	Physiotherapist	0	0	0	6	27	0	0	0	19	0.00
Secondary care											
	Accident & emergency for non psychiatric care	5	22	0	0	0	0	−5	−5	−15	0.34
Mental health service		**2005**	**4930**	**93**	**1622**	**3016**	**84**	**−382**	**2236**	**−3000**	**0.77**
	CRHTT psychiatric visits	20	87	1	0	0	0	−20	19	−59	0.53
	CRHTT nurse visits	6	27	0	24	83	1	18	58	−22	0.38
	CRHTT OT visits	0	0	0	19	82	1	19	57	−19	0.31
	CMHT psychiatric visits	106	299	5	33	83	2	72	647	−71	0.32
	CMHT nurse visits	18	77	1	9	40	0	−8	31	−48	0.68
	CMHT OT visits	10	34	0	39	89	2	29	72	−15	0.20
	AOT social worker visits	0	0	0	39	164	2	39	114	−37	0.31
	AOT OT visits	0	0	0	3	14	0	3	10	−3	0.31
	Psychologist visits	0	0	0	68	230	4	68	174	−39	0.21
	Other doctor visits	7	29	0	0	0	0	−7	6	−20	0.34
	Drug & alcohol advisor visits	16	59	1	0	0	0	−16	10	−43	0.26
	Peer support worker visits	9	6	0	0	0	0	−9	−6	−12	0.00
	Other counselor/therapist visits	0	0	0	18	54	1	18	−7	43	0.16
	Care co-ordinator	6	27	0	10	41	1	4	26	−19	0.75
	Housing support worker	8	33	0	16	67	1	8	43	−26	0.63
	Community mental health centre visits	18	78	1	0	0	0	18	17	−53	0.34
	Daycare centre/day hospital visits	205	750	10	338	840	18	133	647	−381	0.61
	Telephone crisis line calls	0	0	0	6	18	0	6	15	−2	0.14
	Psychiatric admission	1648	4981	77	965	3001	50	−684	1950	−3318	0.61
Total cost to health service		**2136**	**4919**	**99**	**1922**	**3046**	**100**	**−213**	**2408**	**−2835**	**0.87**
Total cost to criminal justice system		18	60	1	0	0	0	−18	9	−45	0.22
	Police contact	4	19	0	0	0	0	−4	4	−13	0.34
	Police doctor	13	58	1	0	0	0	−13	13	−40	0.34
Total cost per case		**2154**	**4919**	**100**	**1922**	**3046**	**100**	**−231**	**−2853**	**2390**	**0.86**

#### Results of cost utility analysis using cost data and clinical outcome data

Initially using point estimates (such as means) from the cost and effect distributions to provide estimates of the cost and effect of the alternative treatments in the primary analysis is valid. However some health economists have warned against separate and sequential hypothesis tests on differences in effects and costs, where there is no statistically significant difference in either, as in this study, which can lead to a superficial observation of the data and an acceptance of a Cost Minimisation Analysis [39]. This could allow researchers to fall into the potential trap of a type II error (a failure to reject the null hypothesis of no difference when in fact a difference does exist). Instead it is suggested that the goal of economic evaluation should be more than hypothesis testing and cost minimisation and should estimate parameters of uncertainty [39]. In other words economic evaluation should estimate the incremental cost effectiveness, with an analytic focus on the estimation of the joint density of cost and effect differences. This, they assert, allows for the quantification of uncertainty, using confidence intervals surrounding the incremental cost-effectiveness ratio (differences in costs between arms divided by the differences in effect between arms) with the presentation of such data as cost-effectiveness acceptability curves. This was achieved in this study by repeat re-sampling from the costs and effectiveness data using non-parametric bootstrapping to generate a distribution of mean costs and effects for the two treatment conditions [40]. These distributions were used to plot the cost effectiveness acceptability curves, which show the probability that Peer Support could be more cost effective compared with standard care for a range of maximum monetary values (ceiling ratios, λ) that a decision maker might be willing to pay [41]. These acceptability curves illustrate the uncertainty associated with the estimate of costs and effects as a result of sampling variation. They were developed as a way of overcoming the statistical difficulties in calculating confidence intervals around incremental cost effectiveness ratios [42,43].

The incremental cost effectiveness ratio (ICER) for utility based on an improvement change in Becks Hopelessness Scale was 12,555. This means that for a 0.02 utility improvement, decision makers might be expected to pay £231.

**Figure 2:**
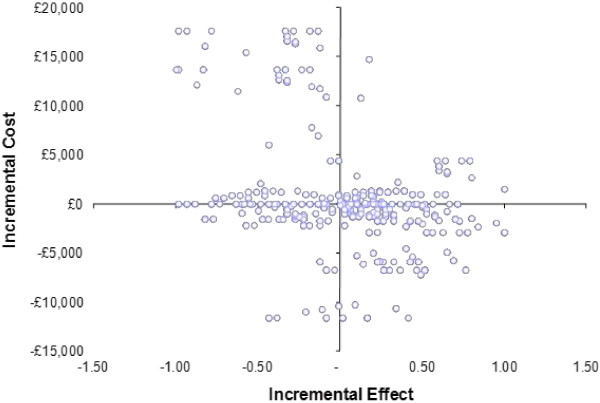
Cost effectiveness plane showing distribution of 1000 replicates of cost and effects for peer support and care as usual using a change in Becks Hopelessness Scale to determine utility scores.

Figure 2 shows the methods described above in order to produce non-parametric bootstrapping to generate a distribution of mean costs and effects for the two treatment conditions using Becks Hopelessness Scale as a measure of effect.

**Figure 3:**
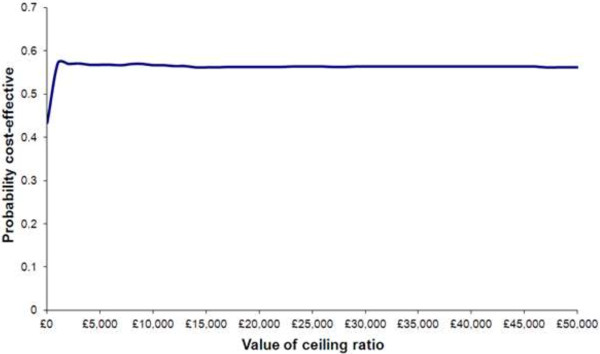
Probability of PS being cost effective given different thresholds for expenditure (using a positive change in Beck’s Hopelessness Scale as a measure of clinical effectiveness).

Figure 3 shows the probability of PS being cost effective given different thresholds for expenditure (using a positive change in Becks Hopelessness Scale as a measure of clinical effectiveness). The ICER for utility based on a change in the EQ5D Scale was -£1,158. This means that despite a utility loss of on average of 0.20 decision makers would still be paying £231 on average.

**Figure 4:**
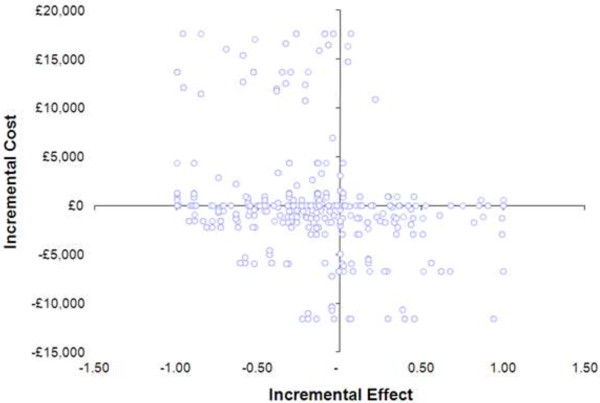
Cost effectiveness plane showing distribution of 1000 replicates of cost and effects for PS and care as usual using the EQ5D.

Figure 4 shows the methods described above in order to produce non-parametric bootstrapping to generate a distribution of mean costs and effects for the two treatments using EQ5D as the outcome measure.

**Figure 5:**
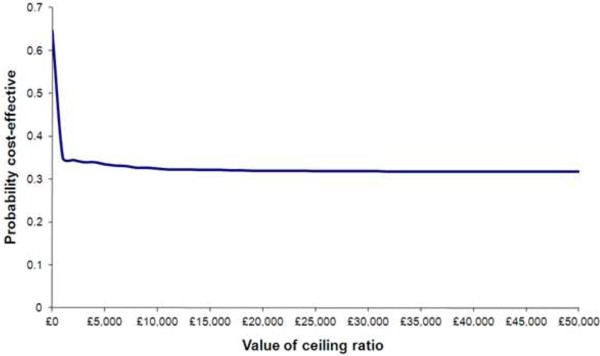
Probability of PS being cost effective given different thresholds for expenditure (using change in EQ5D as a measure of clinical effectiveness).

Figure 5 shows the probability of PS being cost effective given different thresholds for expenditure (using change in EQ5D as a measure of clinical effectiveness).

The cost-effectiveness of PS compared to CAU, using the EQ5D as a measure of utility cannot be demonstrated in this study. This is represented in Figure 4 where the replicates of costs and effects can be seen to fall across all the quadrants on the cost effectiveness plane. Ideally a study depicting cost effectiveness would show most replicates falling in the south east quadrant where costs are lower and the effect of the intervention is greater. In contrast the cost effectiveness plane in Figure 2 shows a distribution of 1000 replicates of cost and effects for PS and care as usual using a change in Beck’s Hopelessness Scale to determine utility scores. This shows a larger number of replicates in the south east quadrant and signifies the potential for the data to show lower costs and higher effects associated with the intervention.

The cost effectiveness acceptability curve in Figure 3 shows that the probability of PS being cost effective given different thresholds for expenditure (using the Becks Hopelessness Scale as a measure of effect), reaches 57% at a given point and exceeds 55% for most of the different potential cost values a decision maker would be willing to spend on the intervention. This was irrespective of how the missing data was handled which may suggest that this data was missing at random and not particularly sensitive to a trend within it.

### Other sensitivity analyses

A re-analysis of the data to exclude potentially expensive non psychiatric drugs was also performed though again this did not make any difference to the overall costs for drugs. Given questions around the cost of providing a PS service, four further scenarios were considered for sensitivity analysis, which were: 1. Conflating costs to a higher overall rate of £540 for the whole period irrespective of hours contributed (as opposed to just paying PSWs expenses as they arose), thus reflecting the total sum of money available to pay service users fulfilling the PS function. 2. Basing costs associated with PSWs on an overall average number of hours. This would be based on the minutes recorded as part of the study for all PSWs and then averaged as an intervention cost across all of the participants in the intervention arm. 3. Using the minimum wage for 2010 (£5.80 per hour) as a proxy for cost rather than expenses. 4. Using the top of a United Kingdom National Health Service Agenda for Change band 3 as a staff cost [44]. This would represent a proxy cost for unqualified staff in the event of a PSW being paid as a salaried member of staff. Overall, running different scenarios for calculating costs around the PSW role did not make any difference to the intervention in terms of its cost or cost-effectiveness. For any of the above scenarios the cost per case for PSWs remains low and in fact hardly differs.

Boot strapping and cost effectiveness acceptability curves were also generated for the cost data that was missing using the mean imputation method, though these did not show any difference in distributions to the previous analyses, with the cost effectiveness acceptability curve being unchanged.

## Discussion

Forty-six service users were successfully recruited to the study against a target of 110; 6.3 per cent of the total number of discharges (n = 734) and 34.3 per cent of the 134 patients approached following prior exclusion of those that did not match our inclusion criteria. Recruitment was affected by a number of factors. Significant delays in obtaining criminal records bureau (CRB) checks for the PSWs led to limited availability of PSWs at the start of the study so recruitment was stalled. Delayed discharges to a number of patients recruited to the study meant that PSWs were ‘tied-up’ with patients still on wards so delaying the recruitment of more patients. Additionally, the study took place in an inner London borough renowned for both its high level of morbidity and a patient population with significant forensic histories. Consequently, a high proportion of patients did not meet inclusion criteria, particularly around perceived severe risk to others (as the PSWs would be lone workers). Additionally a number of patients declined to take part unless they could be guaranteed peer support.

Follow-up rates were disappointing which may again reflect the complex and transient nature of the patient population. Some may have withdrawn in disappointment at being allocated to care-as-usual. More pro-active and creative measures will need to be employed in future studies [45,46].

No significant differences were found between those receiving peer support and those receiving care-as-usual on two of the three main outcome measures of hopelessness and loneliness. However, hope increased in both conditions with a near significant change on Beck’s Hopelessness Scale in those receiving PS. No significant differences between time points or conditions was detected on the EQ-5D quality of life measure, although the EQ VAS general health state scores suggested a significant difference in general health scores between baseline and three-month follow-up for the care-as-usual arm. No immediate explanation for this is available.

Fewer readmissions were reported in the PS arm of the study but no conclusions can be drawn from such a small sample and short follow-up period. Face-to-face contact was made by PSWs about once a week with additional support provided via frequent telephone contact. Studies with different patient groups have reported the successful use of telephone peer support [47,48] and this could be explored further. Future studies could test a more directed or manualised approach with PSWs detailed to make a minimum number of contacts compared with a more flexible patient-empowered approach in line with a recovery focus [49]. Fairly limited time was spent in contact with mental health staff and other agencies. This perhaps reflects the patient and community focus of the intervention but may also echo the indifference shown by many ward staff, as reported by the PSWs on some wards.

This study showed that providing PS is not an expensive intervention and it may be possible to demonstrate cost effectiveness in a larger study. This pilot suggests that although Peer Support has not shown cost effectiveness from a statistical perspective, decision makers could well be willing to pay an extra £231 per person over a three month post discharge period (based on the analysis of resources used), to see a modest improvement change in Beck’s measure of hopelessness. There are also positive wider economic implications for the Peer Support Workers themselves, such as employment and its associated indirect benefits. Employment is a major factor in promoting the social inclusion of people with mental illness [50]. This project, though a modest pilot, did provide a ‘route to employment’ for people with a history of mental illness, in a deprived area where only 8% of people with severe mental illness are in paid work [51]. This study did not explore this and future research may wish to develop this focus.

Previous research suggests that a PS intervention would be successful and potentially cost-effective [17], with claimed savings of half a million Canadian dollars with a care model designed to assist individuals with prolonged psychiatric hospitalization to successfully integrate back into the community. However, the authors do not disclose how the costing methodology was deployed or how these cost savings were calculated. A thorough cost-effectiveness analysis as evidenced in this paper was not carried out, so their claims of overall cost-savings and cost-effectiveness should be viewed with caution.

One earlier study seeking to examine the cost effectiveness of PS at discharge [18] was not shown to be statistically significant, whilst another more recent study [20] did not use prospective RCT methodology but rather a far weaker estimation of potential savings. More recent research in 2013 by the Centre for Mental Health in the UK again highlights that there has been relatively little high quality research into the cost effectiveness of peer support [52]. The report makes the case for peer support being good value for money, especially when the potential for PSWs to reduce psychiatric inpatient bed use, either by preventing admissions or by shortening lengths of stay. The report goes on to argue that because of the very high cost of inpatient care, savings that result from even small changes in bed use may be sufficient to outweigh the costs of employing peer workers. Ultimately the report authors argue that the financial benefits of employing peer support workers do exceed the costs. The authors do however concede that the evidence for their findings is very limited in both quantity and quality, and unlike the results reported in this paper do not arise from trial based data. Asserting the potential for value for money is not the same as showing statistical evidence of cost effectiveness from a properly conducted cost effectiveness study. Cost effectiveness analyses are best designed as part of a randomised controlled trial (RCT) with the rigour that comes from such a methodology with the ability to most likely detect a true statistical cost effect and change. RCTs overall have a lower susceptibility to biased or unfounded random results.

A solid cost effectiveness study of a decent size, demonstrating cost effectiveness of PSWs deployed at discharge has yet to be established, though this pilot provides some very informative data towards the development of a larger study. The authors of this paper would agree with the Centre of Mental Report (52) in supporting a continued interest in the employment of properly trained and supported peer workers in mental health teams, whilst researching more rigorously their effects.

### Limitations and difficulties

Caution needs to be applied to any interpretation of these results given the specific geographical location of the study, the small sample size recruited and the even smaller numbers completing follow-up measures. As a result, this pilot was simply not powered sufficently to detect an effect. Reviews of clinical evaluations have shown how a substantial proportion of studies reporting ‘negative’ results had insufficient power to detect important differences in treatment effect [40,53]. Overall, this reinforces the argument for a larger trial whereby the impact of outliers (and the risk of missing data) will be minimised. This will be especially the case with the cost data where a larger study would have less dramatic overall effects arising from high or zero cost participants.

Missing data for the EQ5D and Becks Hopelessness Scale sometimes exceeded 50%, which resulted in a reliance on mean imputation to calculate all costs. Arguably the use of mean imputation was justifiable given that the alternative in such circumstances might be to default to complete case analysis. However such methods run the risk of insufficient numbers to analyse and as such, are a poorer alternative method and ‘can yield biased estimates’ [54].

There were difficulties in recruiting to the study leading to smaller numbers than the researchers had hoped for. Additionally there was a need to exclude a high number of service users in an inner city environment who had a history of offending and who could not be recruited as participants due to the potential risk to the PSWs who would be lone workers in the community. The study was further complicated by a number of patients being visited by PSWs over an extended period of time whilst still on the ward as their discharge was continually delayed.

These limitations should be considered in the context of the study being a pilot, the purposes of which was to test the feasibility of the study aims and objectives rather than to necessarily show clinical effect or cost-effectiveness at this stage. However there are also counter arguments justifying small sized pilots. For example when undertaking a pilot trial there is often no prior information on which to base a sample size. In such cases the recommendation for pilot studies has been a sample size as little as 12 per group; the justification for this small sample size being that pilots are about testing feasibility and other researchers can use information from the pilot to aid their design of large studies in the future [55].

## Conclusion

This pilot trial of peer support for patients discharged from a mental health hospital found no statistically significant clinical or cost benefits compared to those receiving usual care. However, there is an indication that measures of hopelessness as a reliable indicator of suicide risk may be further decreased in those in receipt of peer support. Lessons learned from the various challenges faced in conducting the study suggest that there is merit in conducting further research on established peer support programmes. Greater consideration should be applied to the design of the study; the nature of the patient population to whom support is offered; the length and frequency of the support provided; and the contact between PSWs and established mental health staff.

The intervention was not costly to deliver, however it is important to highlight that these results do not support, suggest or advocate that peer support workers could or should replace nursing, social work or other qualified mental health staff. In addition, the results have to be considered with caution due to the small sample size obtained. The findings provide useful information and a justification to support a larger scale trial of peer support as an adjunct to existing services. The authors would advocate more research into a) the impact of PS on service users/outcomes; b) the impact of PS roles on PSWs; and c) the impact of PS on the professional workforce, alongside well designed trials.

### Research ethics

Research ethics approval was provided by East London and The City Research Ethics Committee Alpha (Ref: 10/H0704/9). Research governance approval was also obtained from the participating NHS Trust.

## Competing interests

The authors declare that they have no competing interests.

## Authors’ contributions

AS conceived, designed and coordinated the study, collected data, interpreted the results and co-authored the manuscript. CF contributed to design of the study, led on economic analysis and co-authored the transcript. JR conducted the analysis and helped interpret results. JQ collected data and helped interpret results. SH co-designed and delivered PSW training, provided supervision and support to PSWs, liaised with ward staff and identified potential participants. CH co-designed and delivered PSW training and facilitated peer group support. LB contributed to design and conduct of the study. RE contributed to design and conduct of the study. PS contributed to the design and conduct of the study. All authors read and approved the final manuscript.

## Pre-publication history

The pre-publication history for this paper can be accessed here:

http://www.biomedcentral.com/1471-244X/14/30/prepub
